# Neonatal screening program for hemoglobinopathies in the city of São Carlos, state of São Paulo, Brazil: analysis of a series of cases

**DOI:** 10.1016/j.rpped.2014.08.001

**Published:** 2015-03

**Authors:** Camila de Azevedo Silva, Letícia Botigeli Baldim, Geiza César Nhoncanse, Isabeth da Fonseca Estevão, Débora Gusmão Melo

**Affiliations:** a Universidade Federal de São Carlos, São Carlos, SP, Brazil; b Secretaria Municipal de Saúde de São Carlos, São Carlos, SP, Brazil

**Keywords:** Hemoglobinopathies, Neonatal screening, Health care assistance

## Abstract

**OBJECTIVE:**

To analyze the neonatal screening program for hemoglobinopathies in São Carlos, Southeast Brazil, by investigating a series of cases in which the screening test was abnormal. More specifically, it was aimed to know the information regarding the neonatal screening received by mothers at the hospital and at primary health care, in addition to information related to genetic counseling.

**METHODS:**

A descriptive study that enrolled 119 mothers, accounting for 73% of all children born between 2010 and 2011 with abnormal results of neonatal screening for hemoglobinopathies. The mothers completed a questionnaire that assessed the information received at hospital and primary health care, and issues related to genetic counseling. Descriptive statistics was performed.

**RESULTS:**

Of the 119 participating mothers, 69 (58%) had children with sickle cell trait, 22 (18.5%) with hemoglobin C trait, 18 (15.1%) with alpha thalassemia trait and, in 10 cases (8.4%), the result was inconclusive. At the hospital, 118 mothers (99.2%) received information about where to go to collect the test and 115 (96.6%) were informed about the correct time to collect the test. Only 4 mothers (3.4%) were informed about which diseases are investigated and the risks of not performing the screening. Seventeen mothers (14.3%) recognized the difference between trait and disease, and 42 (35.3%) considered that a positive screening test could have implications for future pregnancies. In 70 cases (58.8%), the child's physician was not informed about the screening test results.

**CONCLUSIONS:**

The neonatal screening program needs further improvement. In both scenarios investigated, health professionals demonstrated a lack of training in providing information to mothers and families.

## Introduction

Neonatal screening allows the early identification of several congenital diseases that have no symptoms at birth, in order to intervene in its natural course, thus attenuating the clinical consequences. The criteria used for the inclusion of a disease in a neonatal screening program in general follow those proposed by James Wilson and Gunnar Jungner in 1968: the condition being screened must be an important health problem; the natural history of the disease needs to be well known; there must be an identifiable early stage; early treatment should bring greater benefits than at later stages; an adequate test should be developed for the early stage; the test should be acceptable to the population; retesting intervals should be determined; health care services need to be adequate for the extra clinical work resulting from screening; risks, both physical and psychological, should be fewer than the benefits.^1^

In Brazil, in 2001, the Ministry of Health established the National Neonatal Screening Program (PNTN) with the objective of expanding the existing screening program at the time (restricted to phenylketonuria and congenital hypothyroidism), including the identification of other congenital diseases, such as hemoglobinopathies and cystic fibrosis.^2^ Moreover, the PNTN established a comprehensive approach of the subject, involving early detection, increased population coverage, active search for patients, diagnostic confirmation, follow-up and treatment, and the creation of an information system to register patients.^3^

PNTN was designed as a five-step system, usually organized and conducted by the public health system, which has the necessary conditions and authority to carry out universal screening, in which the pediatrician plays an important role.^4^ The first step comprises the screening test itself and it aims at the universal coverage of screening, i.e., that all newborns be tested. Obstetricians and pediatricians are essential at this stage. Parents need to know about the existence of neonatal screening and be counseled beforehand about which diseases will be screened and benefits of early detection, the risks for the newborn who is not submitted to the test, the appropriate age for its performance, the need for subsequent confirmatory tests for those who test positive, the possibility of false-positives, the process of monitoring and receiving the results. The second step comprises the active search, with the monitoring of results and locating the newborn and the family, especially if the results are altered. The third step comprises performing diagnostic tests, which vary according to the disease and often require specialized laboratories. The fourth step concerns the treatment, when necessary. Finally, the fifth step is the periodic evaluation of all previous steps and different system components: validation of tests used, assessing the efficacy of active search and intervention, evaluating the benefits to the patient, the family and society.^4^

The implementation of the PNTN in Brazil was originally designed to occur in phases, according to the level of organization and coverage of each state of the Federation: in phase I, the diseases screened for are phenylketonuria and congenital hypothyroidism; in phase II, screening for hemoglobinopathies is added to the panel; in phase III, screening for cystic fibrosis is added.^4^ In late 2012, the Ministry of Health authorized the increase of the PNTN into a phase IV, which included the screening of adrenal hyperplasia and biotinidase deficiency.^5^ Currently, 18 units of the federation (including São Paulo) and the Federal District are in phase IV of the PNTN, and nine states are in Phase III.^6^

Hemoglobinopathies are a heterogeneous group of more than 100 inherited diseases, mostly autosomal recessive, with more than 1,000 different mutant alleles characterized at the molecular level, responsible for changes in the structure or synthesis of the hemoglobin molecule, represented mainly by hemoglobin S and C (HbS and HbC), as well as alpha and beta thalassemia.^7^ In Brazil, HbS is present mainly in the African descendants, and sickle-cell anemia (homozygous form of HbS) is the most common genetic disease in the country, with an estimated incidence of 1-3:1,000 live births.^8^ It is estimated that there are two million individuals with sickle cell trait (heterozygous HbS), and eight thousand individuals with sickle cell disease, with the Southeast and Northeast regions being the most affected.^9^ Data from the Ministry of Health, in 2012, showed that the incidence of sickle cell anemia was 1:650 in Bahia, 1:1,300 in Rio de Janeiro, and 1:1,400 in Minas Gerais. These are the three most affected states of the federation, with a frequency of sickle cell trait of 1:17, 1:20 and 1:30, respectively.^10^ Neonatal screening is the starting point to implement simple secondary prevention strategies for sickle cell disease, which include parental education, pneumococcal immunization and prophylaxis with penicillin.^11^^,^ 12

In São Carlos, neonatal screening for hemoglobinopathies started in 1999, so when the PNTN was implemented, the city was already in phase II. Located in the geographic center of the state of São Paulo, São Carlos has approximately 220,000 inhabitants, with approximately 2,700 births/year,^13^ and in 2010 its human development index was 0.805, being considered the 28^th^ most developed municipality in the country.^14^ A previous study carried out in the city showed that the PNTN coverage between the years 2007 and 2010 was, on average, 93.6%, higher than the national and state levels.^15^ In general, there is significant heterogeneity in the distribution of PNTN coverage in Brazil, reflecting the economic, social, political, cultural and health care diversity.^16^ In this context, in São Carlos, the current concern should be primarily to evaluate whether subsequent PNTN steps, in addition to the screening test completion, are being adequately performed. 

In this context, the present study aimed to carry out an analysis of a neonatal screening program for hemoglobinopathies in São Carlos, state of São Paulo, by assessing a series of cases whose screening test results was altered. More specifically, it was aimed to know the information about neonatal screening received by mothers in maternity hospitals and primary health care services, in addition to information related to genetic counseling that mothers had. Finally, it aimed to contribute to the creation of a line of comprehensive care for patients with hemoglobinopathies.

## Method

This is a descriptive study, previously approved by the Ethics Committee on Human Research of UFSCar (number: 121,661) and carried out in the year 2013. Mothers of children born in 2010 and 2011 that had an altered result at the screening test for hemoglobinopathies were invited to participate in the study. Participation was conditional on signing the Free and Informed Consent form.

Initially, data on the PNTN were obtained from Epidemiological Surveillance, identifying 81 children born in 2010 and 82 in 2011 with altered results at the screening test for hemoglobinopathies, which totaled 163 eligible mothers for the study.

There was no refusal to participate in the study among the located mothers; however, in 44 cases (27%), the family could not be located and invited to participate in the study. Thus, 119 mothers were located and invited to participate in the study, representing 73% of the total 163 possible participants.

The 119 mothers who agreed to participate were invited to attend the Primary Health Care Units to which the families were referred and interviewed using a standardized questionnaire developed specifically for this research, used as a data collection tool. The questionnaire was applied by two medical undergraduate students, under the supervision of a physician specialized in hematology. The participants' questions were clarified as the questionnaire was filled out. 

The questionnaire consisted of 24 questions, divided into four parts: (a) the first 10 questions were about the skin color of the father, mother and child, and also about the family's socioeconomic status; (b) four questions on information received by the mother in the maternity hospital regarding neonatal screening; (c) three questions on information received by the mother in Primary Health Care services during the screening test collection, and (d) seven questions related to family genetic counseling provided to the mother due to the presence of an altered screening test result in the child. Information about skin color was reported by mothers; the classification of the Brazilian Institute of Geography and Statistics (IBGE) was used to categorize this variable as: white, black, yellow, brown or Brazilian native. Descriptive statistics of the collected data was performed.

## Results

A total of 119 mothers whose children had an altered hemoglobin screening test participated in the study. None of the children participating in the study was sick, all were heterozygous, i.e., they had an abnormal hemoglobin trait. Sixty-nine participants (58%) had children with sickle-cell trait (HbS); 22 (18.5%) with trace C (HbC); 18 (15.1%) with alpha-thalassemia trait, identified by the presence of Hb Bart's; and in 10 cases (8.4%) the result of the screening was inconclusive, i.e., it was not possible to define the hemoglobinopathy pattern, suggesting a possible rare hemoglobin, specifically non-identified by laboratory methods used in the testing (Electrophoresis by Isoelectric Focusing and High Resolution Liquid chromatography).

Table 1 shows the skin color distribution and socioeconomic profile of this population.

**Table 1:** Ethnicity and socioeconomic profile of the families that participated in the study.

	Fathern (%)	Mothern (%)	Childn (%)
Ethnicity			
White	37 (31.1%)	39 (32.8%)	59 (49.6%)
Mixed-race	62 (52.1%)	54 (45.4%)	50 (42.0%)
Black	20 (16.8%)	26 (21.8%)	10 (8.4%)
Level of schooling			
Elementary School	10 (8.4%)	—	
Elementary I (1^st^ to 5^th^ Grades)	19 (15.%)	12 (10.1%)	
Elementary II (6^th^ to 9^th^ Grades)	32 (26.9%)	36 (30.3%)	
High School	54 (45.4%)	69 (57.9%)	
College/University	4 (3.4%)	2 (1.7%)	
Parents’ marital status			
Living together	106 (89.1%)		
Not living together	13 (10.9%)		
*Family income *per capita			
Up to 1/2 minimum wage	45 (37.8%)		
Between 1/2 and 1 minimum wage	62 (52.1%)		
Between 1 and 2 minimum wages	12 (10.1%)		

**Figure 1:**
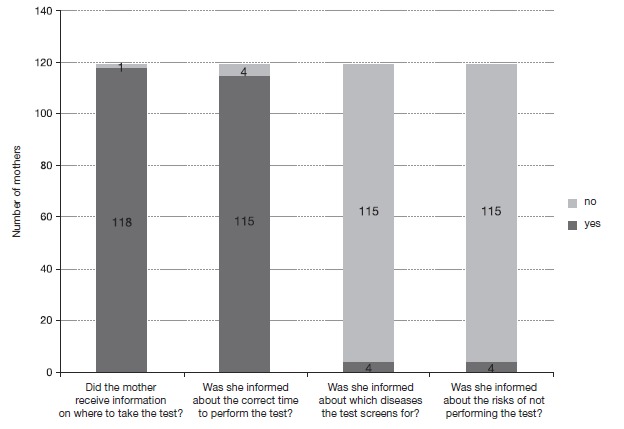
Information received by mothers in the maternity hospital on neonatal screening.

The data shown in Figure 1 reflect the information that mothers received on neonatal screening in the maternity hospital. In only one case (0.8%) the mother reported not having received any information about neonatal screening in the maternity hospital; on the other 111 cases (93.3%) the nurse was the health professional involved in the transmission of this information, with physicians being responsible for only seven cases (5.9%).

**Figure 2:**
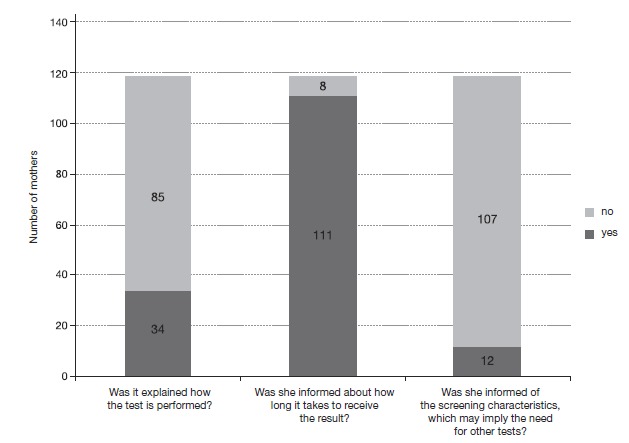
Information received by mothers in Primary Health Care related to the neonatal screening test.

The data shown in Figure 2 translate the information received by mothers in the Primary Health Care (PHC) unit at the time of the neonatal screening test collection. In all 119 cases, the screening test was collected in the first week of life of the newborn. The waiting time for test results averaged 35±42 days, with a median of 30 days, minimum of seven days and a maximum of 110 days. 

**Figure 3:**
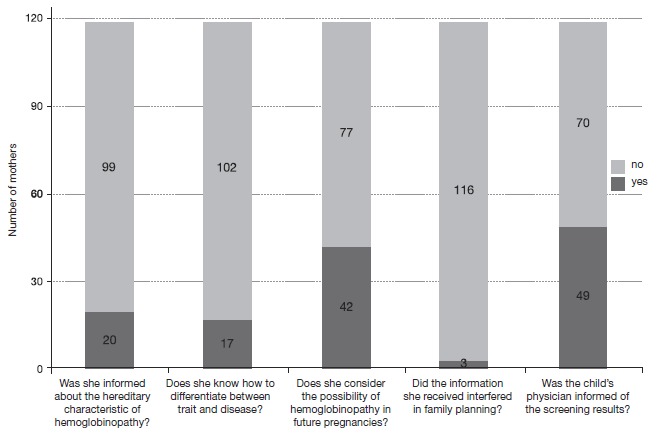
Information related to genetic information that mothers had.

The data shown in Figure 3 refer to information related to genetic counseling that the mothers received, such as information about the hereditary characteristic of hemoglobinopathies, recognizing the difference between trait and disease and the possible implications of the altered test for future pregnancies. In eight cases (6.7%), the families had not returned to the Health Care Unit and were advised in relation to the presence of hemoglobinopathy in the family through this research. As for the other 110 cases (92.5%) the information was provided by the nurse, and in one case (0.8%) the information was provided by a doctor. The frequency of relatives tested was of 72 mothers (60.5%), 17 fathers (14.3%) and 31 siblings (26.1%); the results of tests performed in these families were not available for consultation.

## Discussion

Neonatal screening is currently the best known, and more often used worldwide, public health and preventive pediatric practice related to genetics. In São Carlos, the PNTN coverage between the years 2007 and 2010 was, on average, of 93.6%,^15^ which is greater than the mean national coverage in 2011, which was 83%.^17^ In a study of 30 cities in the state of São Paulo, 57% of municipalities had coverage >90%, 27% of the analyzed cities had coverage between 90 and 80%, 7% between 80 and 70%, and 10% had coverage <70%.^18^ Therefore, the municipality of São Carlos has high coverage in relation to national rates and is in line with other cities in the state of São Paulo. Although the coverage is an essential parameter, one cannot evaluate a neonatal screening program without analyzing the course of care of children with abnormal results and the guidance offered to their families. When proposing the screening, the health system should have the necessary infrastructure to confirm laboratory diagnosis for screened newborns, provide treatment and appropriate counseling; without it, the benefits achieved by early identification are not perpetuated.^16^

Between 2010 and 2011, 163 children with altered results in the neonatal screening for hemoglobinopathies were identified in São Carlos, corresponding to a prevalence of 2.8%, with a prevalence of sickle cell trait, which alone showed a prevalence of 1.2%.

Hemoglobin S and C originated in Africa, propagating widely in the Americas through slave trade.^19^^,^ 20 Thus, the distribution of these hemoglobinopathies is quite heterogeneous in the country, depending on the ethnic characteristics of the population. A study carried out by the Ministry of Health showed that the prevalence of sickle cell trait in the state of Bahia is 5.3%, in Pernambuco and Rio de Janeiro it is 4.0%, in Minas Gerais it is 3.0%, in São Paulo 2.6 %, and in Rio Grande do Sul, 2.0%.^21^ The low prevalence of hemoglobin S and C in São Carlos possibly reflects the ethnic composition of the population, strongly marked by the presence of (mainly Italian) European migration,^22^ so that 73% of the citizens self-reported as being Caucasian in the 2010 Census.^13^

In the present study, we were able to locate and interview 73% of the 163 identified mothers. Address errors were the main obstacle to locating households, reflecting a certain lack of capacity by the Basic Health Unit (BHU) to organize and maintain updated records of their clients. In San Carlos, the collection of biological material (blood) for screening is performed in the BHUs. This material is stored in filter paper and sent to Epidemiological Surveillance, which sends the material to the Associação dos Pais e Amigos dos Excepcionais de São Paulo (APAE-SP), where laboratory tests are performed. The State of São Paulo has four Referral Services for Neonatal Screening (SRTN), accredited with the PNTN and treating patients from the Brazilian Public Health Care Network (SUS), with APAE-SP being the main Neonatal Referral Screening Service, responsible for 64% of all tests performed in the public health care network.^23^ There is no direct communication routine between APAE-SP and the BHUs of the municipality, with this dialogue being intermediated by Epidemiological Surveillance.

A study carried out in Natal, Rio Grande do Norte, based on 1,940 umbilical cord blood samples from newborns of three maternity hospitals in the city, identified 37 cases of abnormal hemoglobin (prevalence of 1.9%), of which 29 (1.5%) had sickle cell trait (HbS), six (0.31%) had C trace (HbC), one (0.05%) had sickle cell anemia (HbSS) and one (0.05%) had Hb Bart's (suggesting alpha thalassemia trait). In this study, correspondence was sent to the newborns' mothers, inviting them to attend a specialized, outpatient clinic and providing them genetic counseling. Of the 37 children identified, only 10 (27.2%) returned for diagnostic confirmation and family investigation. It was presumed that the return consultation was hindered by difficulties locating the address, lack of sufficient knowledge about the importance of the diagnosis of these genetic alterations, and the reduced purchasing power of most families.^24^

A study carried out in the city of Marília, São Paulo, between the years 2004 and 2006, evaluated the performance of the heel prick test, the medical follow-up and prophylactic measures offered, including treatment of children diagnosed with sickle cell disease. During this period, the coverage of the municipality for neonatal screening was of 96.7%, and, as in our study, data collection ran into difficulties related to lack of information on patient follow-up by the HBU. In only one of six cases of sickle cell disease identified in the sample, the patient was correctly referred to a specialized center. In another case, before the child's diagnosis was known, they were hospitalized with complications due to lack of communication between the laboratory that had performed the screening test and the BHU that had not yet received the result and warned the family.^25^ These results are similar to ours, as we also observed a lack of communication between different sectors of the health care system and identified the need to establish protocols and continuing education of health professionals in relation to PNTN. 

We observe that the maternity hospital has partially fulfilled its role to advise on neonatal screening. The BHU performs the collection as recommended, but it does not complete all subsequent stages of PNTN efficiently, even having difficulty locating children whose screening test was altered, which explains the fact that, in eight cases (6.7%), the parents were informed of the presence of hemoglobinopathy in the family through this study. Longitudinal care is not established, as the pediatrician or family doctor does not always know the result of the child's neonatal screening. Information related to genetic counseling is transmitted in a contingent, non-systematic way, and probably because of that many mothers cannot discern between the condition of heterozygosity ("trait") and homozygosity (disease), and do not recognize the possible implications of the presence of hemoglobinopathy in the family.

The misperception between disease and trait is one of the severe problems found in neonatal screening programs for hemoglobinopathies. Ramalho et al reported on the distress of parents of newborns who received the letter from the Neonatal Screening Service stating that the child had a "trait", as they understood that the child had an intellectual disability or other relevant clinical finding.^24^ It is very important that patients, health professionals and the community in general understand that the condition of "trait" (either sickle cell, C or thalassemia trait) is not a disease and it is not an attenuated or incubated form of anemia, which can transform into the disease under certain circumstances. The sickling of red blood cells in individuals with sickle cell trait is rare, occurring only in situations of very intense hypoxia and/or acidosis.^3^^,^ 8

A study carried out in the city of Dourados, state of Mato Grosso do Sul, assessed the efficiency of PNTN for hemoglobinopathies in the years 2001-2005, by analyzing the program's coverage, prevalence of alterations, follow-up of altered cases and the understanding of the families regarding explanations about the disease and genetic counseling. This study found 242 children with altered test results for hemoglobinopathies and the mothers of 32 of them were selected to answer a questionnaire. The results showed 81.4% of neonatal screening coverage in this municipality. Of the 32 assessed families, 20 children (62.5%) were retested and 29 (90.6%) were referred to specialized medical services. These same 29 (90.6%) received requests for blood tests in family members, and in 25 cases (86.2%) the blood collection was performed in fathers and mothers, whereas in four cases (13.8%) only in the mother. The parents' understanding about the genetic counseling information was divided into satisfactory (when there was understanding about heterozygosity), which accounted for 16 cases (55.2%), and unsatisfactory, which amounted to 13 cases (44.8%). There was a positive correlation between illiteracy and poor understanding.^26^ In São Carlos, the line of care for individuals with hemoglobinopathies is organized from the BHU. There is no specialized service specifically for this kind of health care. This might explain why, although the coverage of PNTN is higher in São Carlos that in Dourados, the frequency of tested family members and the level of understanding about the hereditary nature of the condition were lower. 

The Ministry of Health admits the possibility of testing parents and siblings, after the identification of a heterozygous child,^3^ thus recognizing one of the additional benefits of neonatal screening, which is to allow the investigation and counseling of other family members.^27^ It is part of a health strategy, which believes that the identification of heterozygotes may have, secondarily, preventive effects.^28^ The Brazilian proposal is to identify individuals at risk before they (re)start their reproductive plans, in order to inform them about the likelihood of generating sick children in the future, thus expecting them to base their premarital choices or reproductive decisions on this information. We presume that the understanding of how inheritance contributes to the disorder and the risk of recurrence would interfere with the establishment of reproductive associations and family planning.^29^

To some extent, the identification of heterozygotes in the neonatal screening programs for hemoglobinopathies brings tension to genetic disease prevention policies and promotion of fundamental rights (especially the right to reproductive autonomy) and it characterizes what Diniz and Guedes^30^ called the "new genetics". The new genetics believes it controls the revival of eugenics authoritarianism by appealing to the ethical principles of human rights culture, such as individual autonomy, moral pluralism and tolerance. The use of genetic information based on the health rationale is a novelty that creates concerns to the humanist identity of this new discipline. Until recently, this duality of the new genetics was reasonably controlled by the restriction of genetic information to genetic counseling sessions, i.e., for the privilege of clinical genetics, of which object is individual or family-related at maximum, at the expense of population genetics, which intervenes on a community. Currently, the increase in initiatives of community genetics, such as the PNTN, has brought clinical genetics closer to public health^30^, and for this reason, some authors consider that one of the great challenges of the new genetics is to ensure the moral credibility of the discipline.^31^

Regarding genetic counseling, there is also the challenge of ensuring that the content of information provided to families are actually assimilated by them, thus guaranteeing the understanding of the condition. An important part of this function should be to explain the difference between having the disease and having the trait, the transmission of the alteration, where it comes from and the possibilities of affected offspring, both for parents and for the children, explaining to them about the possible combinations of genes in an understandable and ethical way.^30^ In our study, we observed that little of this information was provided to those responsible for the child, and when the health education process was performed, it was carried out as a contingency, and not as routine. 

The Ministry of Health recommends that as the services of neonatal screening increase their population coverage and the spectrum of screened disorders, they must include a clinical geneticist performing genetic counseling of these families and coordinating what the Ministry called "Genetic Counseling in the Scope of the Population Screening Program"^3^ Due to the scarcity of medical geneticists,^32^ this recommendation is not followed in most neonatal screening services in the country.

The municipality of São Carlos has a high neonatal screening coverage, but subsequent stages of the program need improvement. In both scenarios investigated (maternity hospital and BHU), health professionals need training to advise mothers and families. In addition, the Health Units that comprise the BHUs need to ensure an adequate record of users, allowing cases to be recalled for new tests faster. In situations where the screening results indicated alterations, families need to be better informed about genetic counseling and follow-up.

A possible limitation of this study is related to the memory bias, as many investigated aspects were related to past events in the years 2010 and 2011, and there may have been recall errors by the interviewed mothers. Furthermore, because this is a study of a series of cases, there is no control group for comparison.

Hemoglobinopathies represent a genetic disorder with high prevalence and their screening is the first step in prevention. Neonatal screening coverage is increasing in numbers, year by year, and gaining importance as public policy; however, if the next steps of the PNTN do not increase at the same rate, the detection of initially abnormal tests loses meaning.
