# Supplementary material for: Neonatal screening program for hemoglobinopathies in the city of São Carlos, state of São Paulo, Brazil: analysis of a series of cases
Source: Rev Paul Pediatr. 2015 Mar;33(1):19–27. doi: 10.1016/j.rpped.2014.08.001 (PMC4436952; doi:10.1016/j.rpped.2014.08.001)
Supplement: Translation

# Translation: Triagem neonatal de hemoglobinopatias no município de São Carlos, São Paulo, Brasil: análise de uma série de casos

**Authors:** Camila de Azevedo Silva, Letícia Botigeli Baldim, Geiza César Nhoncanse, Isabeth da Fonseca Estevão, Débora Gusmão Melo

**Affiliations:** a Universidade Federal de São Carlos (UFSCar), São Carlos, SP, Brasil; b Secretaria Municipal de Saúde de São Carlos, São Carlos, SP, Brasil

**Keywords:** Hemoglobinopatias, Triagem neonatal, Assistência à saúde

## Abstract

**OBJETIVO:**

Fazer uma análise do programa de triagem neonatal de hemoglobinopatias no município de São Carlos, São Paulo, Brasil, por meio da investigação de série de casos cujo resultado do teste de rastreio foi alterado. Objetivou-se conhecer as informações a respeito da triagem neonatal recebidas pelas mães na maternidade e na atenção primária à saúde, além das informações relacionadas à orientação genética.

**MÉTODOS:**

Estudo descritivo, no qual participaram 119 mães cujos filhos apresentaram teste de triagem de hemoglobinopatia alterado, o que correspondeu a 73% das crianças nascidas entre 2010 e 2011 com resultado de triagem neonatal para hemoglobinopatia anormal. As mães responderam um questionário que avaliou informações recebidas na maternidade e na atenção primária à saúde, além de aspectos relacionados à orientação genética. Foi feita estatística descritiva dos dados.

**RESULTADOS:**

Das 119 mães participantes, 69 (58%) tinham filhos com traço falciforme, 22 (18,5%) traço C, 18 (15,1%) traço alfatalassêmico e 10 (8,4%) resultado inconclusivo. Na maternidade, 118 mães (99,2%) receberam informação sobre onde ir e 115 (96,6%) foram orientadas sobre o momento correto para coleta do teste. Somente quatro mães (3,4%) foram informadas sobre quais doenças seriam investigadas e os riscos de não fazer o rastreio. Das 119 mães participantes, 17 (14,3%) reconheceram a diferença entre traço e doença e 42 (35,3%) consideraram que um teste alterado poderia ter implicações para futuras gestações. Em 70 casos (58,8%), o médico da criança não foi informado sobre o resultado da triagem.

**CONCLUSÕES:**

O programa de triagem neonatal necessita de aperfeiçoamento. Nos dois cenários investigados, os profissionais de saúde carecem de treinamento para orientar mães e famílias.

## Introdução

A triagem neonatal permite a identificação precoce de diversas doenças congênitas que não apresentam sintomas ao nascimento, com a finalidade de intervir no seu curso natural e amenizar as repercussões clínicas. Os critérios usados para inclusão de uma doença em um programa de triagem neonatal, em geral, seguem aqueles propostos por James Wilson e Gunnar Jungner, em 1968: a condição a ser triada deve ser um problema importante de saúde; a história natural da doença precisa ser bem conhecida; deve existir um estágio precoce identificável; o tratamento precoce deve trazer benefícios maiores do que em estágios posteriores; um teste adequado deve ser desenvolvido para o estágio precoce; o teste deve ser aceitável pela população; os intervalos para repetição do teste devem ser determinados; a provisão dos serviços de saúde precisa ser adequada para a carga extra de trabalho clínico resultante da triagem; os riscos, tanto físicos quanto psicológicos, devem ser menores do que os benefícios.^1^

No Brasil, em 2001, o Ministério da Saúde instituiu o Programa Nacional de Triagem Neonatal (PNTN) com o objetivo de ampliar o rastreio existente na época (restrito às doenças fenilcetonúria e hipotireoidismo congênito), incluindo a identificação de outras doenças congênitas, como as hemoglobinopatias e a fibrose cística.^2^ Além disso, o PNTN lançou fundamentos para uma abordagem ampla da questão, que envolveu detecção precoce, ampliação da cobertura populacional, busca ativa de pacientes, confirmação diagnóstica, acompanhamento e tratamento adequados, além da criação de um sistema de informações para cadastrar os doentes.^3^

O PNTN foi concebido como um sistema de cinco etapas, habitualmente organizado e conduzido pelo sistema público de saúde, que tem as condições e a autoridade necessárias para execução da triagem universal, na qual o pediatra desempenha um papel importante.^4^ A primeira etapa compreende o teste de triagem propriamente dito e objetiva a cobertura universal do rastreio, ou seja, que todos os recém-nascidos sejam testados. As atuações do obstetra e do pediatra são fundamentais nessa fase. Os pais precisam saber da existência da triagem neonatal e ser orientados previamente sobre quais doenças serão triadas e os benefícios da detecção precoce; os riscos existentes para o recém-nascido que não é submetido ao teste; a idade adequada para sua feitura; a necessidade de exames confirmatórios posteriores para os que forem positivos; a possibilidade de falso-positivo; o processo de acompanhamento e recebimento dos resultados. A segunda etapa compreende a busca ativa, com acompanhamento do resultado e localização do recém-nascido e sua família, principalmente se o resultado for alterado. A terceira etapa compreende a feitura de testes diagnósticos, que variam de acordo com a doença e que, frequentemente, requerem laboratórios especializados. A quarta etapa diz respeito ao tratamento, quando necessário. Finalmente, a quinta etapa é a avaliação periódica de todas as etapas anteriores e dos diferentes componentes do sistema: validação dos testes usados, verificação da eficiência da busca ativa e intervenção, verificação do beneficio para o paciente, a família e a sociedade.^4^

A implantação do PNTN no Brasil foi originalmente desenhada para ocorrer em fases, de acordo com o nível de organização e cobertura de cada estado da federação: na fase I, as doenças triadas são a fenilcetonúria e o hipotireoidismo congênito; na fase II, é adicionada ao painel a triagem para hemoglobinopatias; na fase III, acrescenta-se a triagem para fibrose cística.^4^ No fim de 2012, o Ministério da Saúde autorizou a expansão do PNTN para a fase IV, que incluiu o rastreio de hiperplasia adrenal e deficiência de biotinidase.^5^ No momento, 18 unidades da federação (incluindo São Paulo) e o Distrito Federal encontram-se na fase IV do PNTN e mais nove estados estão na Fase III.^6^

As hemoglobinopatias são um grupo heterogêneo de mais de 100 doenças hereditárias, na maioria autossômicas recessivas, com mais de 1.000 diferentes alelos mutantes caracterizados em nível molecular, responsáveis por alterações na estrutura ou síntese da molécula de hemoglobina, representadas principalmente pelas hemoglobinas S e C (HbS e HbC) e pelas alfa e beta talassemias.^7^ No Brasil, a HbS está presente sobretudo na população negra e a anemia falciforme (forma homozigótica da HbS) é a doença genética mais frequente no país, com incidência estimada em 1-3:1.000 nascidos vivos.^8^ Estima-se a existência de dois milhões de indivíduos com o traço falciforme (heterozigotos para HbS) e oito mil com doença falciforme, as regiões Sudeste e Nordeste são as mais acometidas.^9^ Dados do Ministério da Saúde de 2012 mostram incidência de anemia falciforme de 1:650 na Bahia, 1:1.300 no Rio de Janeiro e 1:1.400 em Minas Gerais. Esses são os três estados da federação mais acometidos, com frequência de traço falciforme de 1:17, 1:20 e 1:30, respectivamente.^10^ A triagem neonatal é o ponto de partida para estratégias simples de prevenção secundária da doença falciforme, que incluem educação dos pais, imunização antipneumocócica e profilaxia com penicilina.^11^ ^and^ 12

Em São Carlos, a triagem neonatal para hemoglobinopatias foi iniciada em 1999. Assim, quando o PNTN foi implantado, a cidade já estava na fase II. Localizada no centro geográfico do Estado de São Paulo, São Carlos tem cerca de 220.000 habitantes, aproximadamente 2.700 nascimentos/ano^13^ e em 2010 apresentou índice de desenvolvimento humano de 0,805 e foi considerado o 28° município mais desenvolvido do país.^14^ Estudo anterior, feito na cidade, mostrou que a cobertura do PNTN entre 2007 e 2010 foi, em média, 93,6% superior aos índices nacionais e estaduais.^15^ De forma geral, há expressiva heterogeneidade na distribuição da cobertura do PNTN no Brasil, o que reflete a diversidade econômica, social, política, cultural e de saúde.^16^ Nesse contexto, em São Carlos, a preocupação atual deve ser, principalmente, avaliar se as etapas subsequentes do PNTN, para além da feitura do teste de triagem, estão sendo adequadamente efetivadas.

O presente estudo teve como objetivo fazer uma análise do programa de triagem neonatal de hemoglobinopatias no município de São Carlos, São Paulo, por meio da investigação de uma série de casos cujo resultado do teste de rastreio foi alterado. Mais especificamente, objetivou-se conhecer as informações a respeito da triagem neonatal recebidas pelas mães na maternidade e na atenção primária à saúde, além das informações relacionadas à orientação genética que as mães tinham. Em última instância, pretendeu-se colaborar para construção de uma linha de cuidado integral para pacientes com hemoglobinopatias.

## Método

Trata-se de estudo descritivo, previamente aprovado pelo Comitê de Ética em Pesquisa com Seres Humanos da UFSCar (parecer 121.661) e conduzido durante 2013. Foram convidadas a participar da pesquisa as mães das crianças nascidas em 2010 e 2011 que apresentaram teste de triagem de hemoglobinopatia alterado. A participação foi condicionada à assinatura de Termo de Consentimento Livre e Esclarecido.

Inicialmente, foi feito levantamento dos dados do PNTN na Vigilância Epidemiológica, com identificação de 81 crianças nascidas em 2010 e 82 em 2011 com teste de triagem de hemoglobinopatias alterado, o que totalizou 163 possíveis mães participantes da pesquisa.

Entre as mães localizadas, não houve recusa de participação. Contudo, em 44 casos (27%) não foi possível localizar a família e convidá-la para o estudo. Assim, foram localizadas e incluídas no estudo 119 mães, o que correspondeu a 73% do total de 163 possíveis participantes da pesquisa.

As 119 mães que concordaram em participar do estudo foram convidadas a comparecer nas unidades de atenção primária à saúde às quais suas famílias estavam adscritas e entrevistadas por meio de questionário padronizado, elaborado especificamente para esta pesquisa, e usado como instrumento de coleta de dados. O questionário foi aplicado por duas estudantes de graduação em medicina, sob supervisão de uma médica hematologista. Durante o preenchimento do questionário as dúvidas apresentadas pelas participantes foram esclarecidas.

O questionário, era composto por 24 perguntas, divididas em quatro partes: (a) 10 primeiras perguntas sobre cor da pele do pai, da mãe e da criança e também sobre condições socioeconômicas da família; (b) quatro perguntas sobre informações recebidas pela mãe na maternidade a respeito da triagem neonatal; (c) três perguntas sobre informações recebidas pela mãe na Atenção Primária à Saúde durante a coleta do teste de triagem; (d) e sete perguntas relacionadas a orientação genética familiar fornecida à mãe, mediante a presença de teste de triagem alterado em seu filho. As informações sobre cor de pele foram declaradas pelas mães; para categorizar essa variável foi usada a classificação do Instituto Brasileiro de Geografia e Estatística (IBGE): branca, preta, amarela, parda ou indígena.

Foi feita estatística descritiva dos dados coletados.

## Resultados

Participaram desta pesquisa 119 mães cujos filhos apresentaram teste de triagem de hemoglobinopatia alterado. Nenhuma criança deste estudo era doente, todas eram heterozigotas, ou seja, tinham "traço" para alguma hemoglobina anormal. Sessenta e nove participantes (58%) tinham filhos com traço falciforme (HbS); 22 (18,5%) com traço C (HbC); 18 (15,1%) com traço alfatalassêmico, identificado pela presença de Hb Bart's; e em 10 situações (8,4%) o resultado da triagem foi inconclusivo, ou seja, não foi possível definir o padrão da hemoglobinopatia, o que sugere uma provável hemoglobina rara, não identificada especificamente pelos métodos laboratoriais usados no ensaio (Eletroforese por Focalização Isoelétrica e Cromatografia Líquida de Alta Resolução).

A tabela 1 apresenta a distribuição da cor da pele e o perfil socioeconômico dessa população estudada.

**Tabela 1:** Cor da pele e perfil socioeconômico das famílias que participaram do estudo

	pai	mãe	criança
	n (%)	n (%)	n (%)
*Cor da pele*			
Branca	37 (31,1%)	39 (32,8%)	59 (49,6%)
Parda	62 (52,1%)	54 (45,4%)	50 (42,0%)
Preta	20 (16,8%)	26 (21,8%)	10 (8,4%)

*Escolaridade*			
Elementar	10 (8,4%)	-	
Fundamental I (1° a 5° anos)	19 (15,%)	12 (10,1%)	
Fundamental II (6° a 9° anos)	32 (26,9%)	36 (30,3%)	
Médio	54 (45,4%)	69 (57,9%)	
Superior	4 (3,4%)	2 (1,7%)	

*Estado marital dos pais*			
Vivem juntos	106 (89,1%)		
Não vivem juntos	13 (10,9%)		

*Renda familiar mensal per capita*			
Até 1/2 salário mínimo	45 (37,8%)		
Entre 1/2 e 1 salário mínimo	62 (52,1%)		
Entre 1 e 2 salários mínimos	12 (10,1%)		

Os dados apresentados na figura 1 traduzem as informações que as mães receberam sobre a triagem neonatal na maternidade. Em apenas um caso (0,8%) a mãe relatou não ter recebido qualquer informação sobre a triagem neonatal na maternidade; nos restantes, em 111 casos (93,3%) a enfermeira foi a profissional de saúde envolvida na transmissão dessas informações, os médicos foram os responsáveis em apenas sete casos (5,9%).

**Figura 1:** Informações recebidas pelas mães na maternidade sobre a triagem neonatal.

Os dados apresentados na figura 2 traduzem as informações recebidas pelas mães na Atenção Primária à Saúde (APS), no momento da coleta do teste de triagem neonatal. Em todos os 119 casos, o teste de triagem foi coletado na primeira semana de vida do recém-nascido. O tempo de espera pelo resultado do teste foi em média de 35 ± 42 dias, com mediana de 30 dias, tempo mínimo de sete dias e máximo de 110 dias.

**Figura 2:** Informações recebidas pelas mães na Atenção Primária à Saúde relacionadas ao teste de triagem neonatal.

Os dados apresentados na figura 3 dizem respeito às informações relacionadas à orientação genética que as mães tinham, como: informação sobre o caráter hereditário da hemoglobinopatia, reconhecimento da diferença entre traço e doença e das possíveis implicações do teste alterado para futuras gestações. Em oito casos (6,7%), as famílias não haviam retornado à unidade de saúde e foram orientadas em relação à presença de hemoglobinopatia na família por meio desta pesquisa. Nos restantes, em 110 casos (92,5%,) as informações foram fornecidas pela enfermeira e em um caso (0,8%) os esclarecimentos foram feitos pelo médico. A frequência de parentes testados foi de 72 mães (60,5%), 17 pais (14,3%) e 31 irmãos (26,1%); os resultados dos testes feitos nesses parentes não estavam disponiveis para consulta.

**Figura 3:** Informações relacionadas à orientação genética que as mães tinham.

## Discussão

A triagem neonatal é atualmente a prática de saúde pública e de pediatria preventiva relacionada à genética mais conhecida e usada em todo o mundo. Em São Carlos, a cobertura do PNTN entre 2007 e 2010 foi, em média, de 93,6%,^15^ valor superior à média de cobertura nacional, que em 2011 foi de 83%.^17^ Em pesquisa que envolveu 30 cidades do interior do Estado de São Paulo, 57% dos municípios apresentaram cobertura acima de 90%, 27% das cidades investigadas tiveram cobertura entre 90 e 80%, 7% entre 80 e 70% e 10% inferior a 70%.^18^ Portanto, São Carlos tem cobertura elevada em relação às taxas nacionais e está em consonância com outras cidades do interior do Estado de São Paulo. Apesar de a cobertura ser um parâmetro essencial, não é possível avaliar um programa de triagem neonatal sem analisar o fluxo de atendimento das crianças com resultados alterados e as orientações oferecidas às suas famílias. Ao propor a triagem, o sistema de saúde deve ter infraestrutura necessária para confirmar o diagnóstico laboratorial dos recém-nascidos triados, providenciar o tratamento e o aconselhamento adequados; sem isso, os benefícios obtidos pela identificação precoce não são perpetuados.^16^

Entre 2010 e 2011 foram identificadas 163 crianças com resultado alterado na triagem neonatal de hemoglobinopatias de São Carlos, o que corresponde a uma prevalência de 2,8%, com predomínio do traço falciforme, que sozinho alcançou prevalência de 1,2%.

As hemoglobinas S e C tiveram origem na África, com propagação ampla nas Américas por meio do tráfico de escravos.^19^ ^and^ 20 Dessa forma, a distribuição dessas hemoglobinopatias é bastante heterogênea no país, depende de características étnicas da população. Pesquisa divulgada pelo Ministério da Saúde mostrou que na Bahia a prevalência de traço falciforme é de 5,3%, em Pernambuco e no Rio de Janeiro 4%, em Minas Gerais 3%, em São Paulo 2,6% e no Rio Grande do Sul 2%.^21^ As baixas prevalências das hemoglobinas S e C em São Carlos possivelmente refletem a composição étnica da população, fortemente marcada pela presença de emigração europeia (notadamente italiana),^22^ de tal modo que 73% dos cidadãos se autodeclararam como tendo cor de pele branca no Censo de 2010.^13^

No presente estudo, conseguimos localizar e entrevistar 73% das 163 mães identificadas. Erros de endereço foram o principal obstáculo na localização das famílias, o que refletiu certo despreparo da APS para organizar e manter atualizados registros da sua clientela. Em São Carlos, a coleta do material biológico (sangue) para triagem é feita nas unidades da APS. Esse material é acondicionado em papel-filtro e enviado para a Vigilância Epidemiológica, que o remete à Associação dos Pais e Amigos dos Excepcionais de São Paulo (Apae-SP), local onde são feitos os ensaios laboratoriais. O Estado de São Paulo tem quatro Serviços de Referência de Triagem Neonatal (SRTN), credenciados no PNTN e que atendem o SUS. A Apae-SP é o maior Serviço de Referência de Triagem Neonatal, responsável por 64% de todos os testes feitos na rede pública de saúde.^23^ Não há uma rotina de comunicação direta entre a Apae-SP e as unidades da atenção primária à saúde do município, esse diálogo sempre é intermediado pela Vigilância Epidemiológica.

Pesquisa feita em Natal, Rio Grande do Norte, a partir de 1.940 amostras de sangue de cordão umbilical provenientes de recém-nascidos de três maternidades da cidade, identificou 37 casos de hemoglobinas anormais (prevalência de 1,9%), dos quais 29 (1,5%) tinham traço falciforme (HbS), seis (0,31%) traço C (HbC), um (0,05%) anemia falciforme (HbSS) e um (0,05%) Hb Bart's (o que sugere traço alfatalassêmico). Nessa pesquisa, enviou-se correspondência para as mães dos recém-nascidos, para convidá-las a participar de um ambulatório especializado e oferecer aconselhamento genético. Das 37 crianças detectadas, apenas 10 (27,2%) retornaram para confirmação diagnóstica e investigação familiar. Admitiu-se que o retorno foi prejudicado por dificuldade de localização de endereço, falta de conhecimento suficiente sobre a importância do diagnóstico dessas alterações genéticas e reduzido poder aquisitivo da maioria das famílias.^24^

Estudo feito na cidade de Marília, São Paulo, entre 2004 e 2006, avaliou a feitura do "teste do pezinho", o acompanhamento médico e as medidas profiláticas oferecidas, incluindo tratamento de crianças diagnosticadas com doença falciforme. Nesse período, a cobertura do município para triagem neonatal foi de 96,7% e, assim como no nosso estudo, a coleta de dados esbarrou em dificuldades relacionadas com falta de informação sobre o seguimento dos pacientes pela APS. Somente em um dos seis casos de doença falciforme identificados na amostra o paciente foi devidamente encaminhado para um centro de referência. Em outro caso, antes que se conhecesse o diagnóstico da criança, ela foi internada com complicações da doença por falta de comunicação entre o laboratório que havia feito o exame de triagem e a unidade de saúde que ainda não havia recebido o resultado e avisado à família.^25^ Esses resultados são semelhantes aos nossos, pois também notamos falta de comunicação entre os diferentes setores do sistema de saúde e identificamos necessidade de criação de protocolos e de educação permanente dos profissionais de saúde em relação ao PNTN.

Observamos que a maternidade tem cumprido parcialmente seu papel ao orientar sobre a triagem neonatal. A APS faz a coleta conforme preconizado, mas não concretiza todas as etapas subsequentes do PNTN com eficiência, há até dificuldades para localizar as crianças cujo teste de triagem foi alterado, o que explica que em oito casos (6,7%) os pais tenham sido informados sobre a presença de hemoglobinopatia na família por esta pesquisa. Não se estabelece uma longitudinalidade de cuidado, visto que nem sempre o pediatra ou médico de família da criança sabe o resultado da triagem neonatal. Informações relacionadas à orientação genética são transmitidas de forma contingencial, e não sistemática, e provavelmente por causa disso muitas mães não conseguem discernir entre a condição de heterozigose ("traço") e de homozigose (doença) e não reconhecem as possíveis implicações da presença da hemoglobinopatia na sua família.

A confusão entre traço e doença é um dos problemas graves surgidos nos programas de triagem neonatal de hemoglobinopatias. Ramalho et al. relatam aflição de pais de recém-nascidos que, ao receber carta do Serviço de Triagem Neonatal comunicando que a criança era portadora de "traço", entenderam que a criança era portadora de deficiência intelectual ou de outra alteração clínica relevante.^24^ É muito importante que fique bem claro para pacientes, profissionais de saúde e para comunidade em geral que a condição de "traço" (seja falciforme, seja traço C ou talassêmico) não é uma doença e não é uma forma atenuada ou incubada de anemia, que pode se transformar em doença em determinadas circunstâncias. A falcização de hemácias nos indivíduos com traço falcêmico é excepcional, só ocorre em situações de hipóxia e/ou acidose muito intensas.^3^ ^and^ 8

Estudo feito na cidade de Dourados, Mato Grosso do Sul, avaliou a eficiência do PNTN para hemoglobinopatias de 2001 a 2005, analisou cobertura do programa, prevalência de alterações, acompanhamento dos casos alterados e compreensão das famílias quantos às explicações sobre a doença e o aconselhamento genético. Nesse estudo foram encontradas 242 crianças com teste alterado para hemoglobinopatias e 32 foram sorteadas para que suas mães respondessem um questionário. Os resultados mostraram cobertura da triagem neonatal de 81,4% nesse município. Dentre as 32 famílias investigadas, 20 crianças (62,5%) fizeram reteste e 29 (90,6%) foram referenciadas para serviço médico especializado. Essas mesmas 29 (90,6%) tiveram solicitação para coleta de sangue de parentes e em 25 casos (86,2%) fez-se coleta de sangue de pais e mães e em quatro casos (13,8%) somente da mãe. A compreensão dos pais quanto às informações do aconselhamento genético foi dividida em satisfatória (quando havia entendimento sobre heterozigose), que representou 16 casos (55,2%), e insatisfatória, que equivaleu a 13 entrevistados (44,8%). Houve correlação positiva entre compreensão insatisfatória e analfabetismo.^26^ Em São Carlos, a linha de cuidado para indivíduos com hemoglobinopatias está organizada a partir da APS. Não existe um serviço especializado constituído somente para esse tipo de atendimento. Isso talvez explique por que, apesar de a cobertura do PNTN ser maior em São Carlos do que em Dourados, a frequência de parentes testados e o nível de compreensão sobre o caráter hereditário da condição foram menores.

O Ministério da Saúde admite a possibilidade de testagem nos pais e irmãos, a partir da identificação de uma criança heterozigota,^3^ e reconhece um dos benefícios adicionais da triagem neonatal, que é possibilitar a investigação e o aconselhamento de outros membros da família.^27^ Parte-se de uma estratégia sanitária, que acredita que a identificação de heterozigotos pode ter, secundariamente, efeitos preventivos.^28^ A proposta brasileira é identificar as pessoas em risco antes que (re)iniciem seus projetos reprodutivos, a fim de informá-las sobre a probabilidade de gerarem futuras crianças doentes, na expectativa de elas fundamentarem suas escolhas pré-conjugais ou decisões reprodutivas nessa informação. Aposta-se que a compreensão sobre o modo como a hereditariedade contribui para a doença e o risco de recorrência interferiria no estabelecimento de vínculos reprodutivos e no planejamento familiar.^29^

Em alguma medida, a identificação de heterozigotos nos programas de triagem neonatal de hemoglobinopatias é motivo de tensão entre políticas de prevenção de doenças genéticas e de promoção de direitos fundamentais (especialmente o direito à autonomia reprodutiva) e isso caracteriza o que Diniz e Guedes^30^ denominaram "nova genética". A nova genética acredita controlar o renascimento do autoritarismo eugênico pelo apelo a princípios éticos da cultura dos direitos humanos, tais como a autonomia individual, o pluralismo moral e a tolerância. O uso da informação genética com base em uma lógica sanitarista é uma novidade que provoca inquietações à identidade humanista dessa nova disciplina. Até pouco tempo, essa dualidade da nova genética era razoavelmente controlada pela concentração da informação genética às sessões de aconselhamento genético, ou seja, pelo privilégio da genética clínica, cujo objeto é individual ou no máximo familiar, em detrimento da genética populacional, que intervém sobre uma comunidade. Hoje, o amplo crescimento de iniciativas de genética comunitária, como o PNTN, aproximou a genética clínica da saúde pública^30^ e, por esse motivo, alguns autores consideram que um dos grandes desafios da nova genética é garantir a credibilidade moral da disciplina.^31^

No que diz respeito ao aconselhamento genético, ainda existe o desafio de garantir que o conteúdo e o teor das informações transmitidas para as famílias sejam de fato assimilados, para assegurar entendimento sobre a condição. Parte de destaque nessa função deve ser explicar a diferença entre ter a doença e ter o traço, a transmissão da alteração, de onde ela vem e as possibilidades de descendentes afetados, tanto para os pais quanto para os filhos, e as possíveis combinações de genes de uma forma compreensível e ética.^30^ No nosso estudo, observamos que poucas dessas informações foram repassadas aos responsáveis pela criança e quando o processo de educação em saúde foi feito, o foi de forma contingencial, e não como rotina.

O Ministério da Saúde recomenda que, à medida que os serviços de triagem neonatal aumentem sua cobertura populacional e o seu espectro de patologias triadas, passem a contar com geneticista clínico para fazer o aconselhamento genético dessas famílias e coordenar o que o ministério denominou de Aconselhamento Genético no Âmbito de Programa de Triagem Populacional'.^3^ Em função da escassez de profissionais médicos geneticistas,^32^ essa recomendação não é seguida na maior parte dos serviços de triagem neonatal do país.

São Carlos tem uma alta cobertura de triagem neonatal, porém as etapas subsequentes do programa necessitam de aperfeiçoamento. Nos dois cenários investigados (maternidade e APS), os profissionais de saúde necessitam de treinamento para orientar mães e famílias. Além disso, as unidades de saúde que compõem a APS precisam garantir um registro adequado dos usuários, que permita reconvocar casos para feitura de novos exames com agilidade. Nas situações nas quais o resultado da triagem apontou alterações, as famílias precisam ser mais bem esclarecidas em relação à orientação genética e seguimento dos pacientes.

Uma possível limitação deste estudo está relacionada com o viés de memória, já que muitos aspectos investigados faziam referência a acontecimentos passados em 2010 e 2011 e é possível que existam erros de recordação por parte das mães entrevistadas. Além disso, por se tratar de um estudo de série de casos, não há um grupo controle para comparação.

As hemoglobinopatias representam uma alteração genética com grande prevalência e seu rastreio é a primeira medida de prevenção. A cobertura da triagem neonatal está ano a ano avançando em números e ganhando importância como política pública. Porém, se as etapas seguintes do PNTN não caminharem na mesma proporção, a detecção de testes alterados inicialmente perde o sentido.
